# Endothelin-converting-enzyme 1 inhibition and CGRP receptor recycling in human coronary and middle meningeal arteries

**DOI:** 10.1186/1129-2377-14-S1-P95

**Published:** 2013-02-21

**Authors:** S Labruijere, R De Vries, AHJ Danser, GS Cottrell, A MaassenVanDenBrink

**Affiliations:** 1; 2

## 

Although best known for its role in the conversion of big endothelin to endothelin-1, endothelin-converting enzyme 1 (ECE-1) also regulates the resensitization of certain neuropeptide receptors, including the receptor for calcitonin gene-related peptide (CGRP) ( Padilla et al., 2007 ). We investigated the role of ECE-1 in the resensitization of responses to CGRP in human coronary (HCA) and middle meningeal (HMA) arteries using the potent and selective ECE-1 inhibitor, SM-19712. Segments of HCA (Ø 0.5–1 mm) and HMA (Ø 0.5–1 mm) were mounted in organ baths and concentration response curves (CRCs) to CGRP were constructed in the absence or presence of the ECE-1 inhibitor SM-19712. After the first CRC to CGRP the segments were washed and after 30-45 minutes a second CRC was constructed in the absence or presence of SM-19712 to investigate ECE-1-dependent CGRP resensitization. Furthermore, CRCs to big endothelin were constructed in the presence or absence of SM-19712. In both HCA and HMA, no differences were seen between the initial responses to CGRP in the absence or presence of SM-19712 (HCA E_max+SM19712_ 94**±**8%, E_max_**_–_**_SM19712_ 93**±**5%; pEC_50+SM19712_ 9.1**±**0.2, pEC_50-SM19712_ 9.2**±**0.1; HMA E_max+SM19712_ 72**±**7%, E_max_**_–_**_SM19712_ 59**±**7%; pEC_50+SM19712_ 8.5**±**0.4, pEC_50-SM19712_ 8.1**±**0.8), as well as between the second CRCs to CGRP in the absence or presence of SM-19712 (HCA E_max+SM19712_ 110**±**13%, E_max_**_–_**_SM19712_ 78**±**22%; pEC_50+SM19712_ 7.5**±**0.5, pEC_50-SM19712_ 7.9**±**0.01; HMA E_max+SM19712_ 38**±**13%, E_max_**_–_**_SM19712_ 44**±**1%; pEC_50+SM19712_ 8.6**±**0.5, pEC_50-SM19712_ 7.8**±**0.9). Furthermore, contractions to big endothelin were not different in the absence or presence of SM-19712 in either HCA (E_max+SM19712_ 118**±**14%, E_max-SM19712_ 115**±**32%; pEC_50+SM19712_ 6.0**±**0.5, pEC_50-SM19712_ 6.9**±**0.2) or HMA (E_max+SM19712_ 121**±**1%, E_max_**_–_**_SM19712_ 147**±**19%; pEC_50_**_–_**_SM19712_ 7.4**±**0.4, pEC_50+SM19712_ 7.0**±**0.8). Our results indicate that ECE-1 does not regulate the resensitization of CGRP responses in HCA and HMA.
